# Remineralization of teeth with casein phosphopeptide-amorphous calcium phosphate: analysis of salivary pH and the rate of salivary flow

**DOI:** 10.1038/s41405-023-00141-z

**Published:** 2023-04-11

**Authors:** Lubna Alkarad, Muaaz Alkhouli, Mayssoon Dashash

**Affiliations:** Faculty of Dentistry, Damascus University, Damascus, Syrian Arab Republic

**Keywords:** Paediatric dentistry, Dental public health

## Abstract

**Aim:**

To investigate the changes of salivary flow rate and salivary pH of Syrian children with mixed dentition following application of Casein Phosphopeptide-Amorphous Calcium Phosphate (CPP-ACP).

**Methods:**

This study is part of a double-blind randomized controlled clinical trial. It included 50 children aged 6–8 who were randomly divided into two treatment groups to receive either CPP-ACP GC Tooth Mousse™ (Group A) or placebo (Group B) with 25 participants per group. After the application of the product in the mouth for 3 min, saliva samples were collected four times (T0, T1, T2, and T3) to measure salivary pH and the rate of salivary flow.

**Results:**

There was no significant difference between group A and B in the mean value of salivary flow rate (*t* = 1.08, *P* = 0.28, 0.57 ± 0.28 versus 0.56 ± 0.38 respectively) and salivary pH (*t* = 0.61, *P* = 0.54, 7.28 ± 0.44 versus 7.25 ± 0.36 respectively). However, there was a significant difference between different time points (T0, T1, T2, and T3) in the mean value of salivary flow rate (0.41 ± 0.30, 0.65 ± 0.36, 0.53 ± 0.28, and 0.56 ± 0.34 respectively) and salivary pH (6.99 ± 0.44, 7.46 ± 0.36, 7.36 ± 0.32, and 7.26 ± 0.32 respectively).

**Conclusion:**

The application of the GC Tooth Mouse (CPP-ACP) was similar to placebo in increasing the salivary pH and salivary flow rate.

**Trial registration:**

ISRCTN17509082, Registration date 22/11/2022.

## Introduction

Dental caries is one of the most common multifactorial diseases that occurs in children, due to imbalance between protective and pathological factors [1, 2] causing loss of calcium and phosphate ions as well as demineralization. However, this process can be reversed by the so-called remineralization process [3]. Saliva, fluoride supplements and diet are preventive regimes against tooth demineralization [4]. In addition, several materials have been introduced to inhibit demineralization and enhance the effect of remineralization. such as xylitol, bio-active glass, tricalcium phosphate and CPP-ACP [5, 6].

The CPP–ACP is a bioactive material derived from the milk protein casein [7]. It has been reported to inhibit enamel demineralization by maintaining the supersaturation of calcium and phosphate in saliva, buffering of plaque pH and increasing the calcium and phosphate ions level in plaque [8, 9]. The application of topical CPP-ACP was found to be beneficial for the remineralization process of teeth as it was able to raise salivary pH and salivary flow rate [9, 10].

Several studies have reported that the saliva pH can be changed due to variant factors such as metabolic, hormonal and general health changes [11–13]. However, decreased levels of salivary pH is an important indicator of developing dental caries [14].

Salivary flow rate can be defined as the amount of saliva secreted per minute. It can be considered as an important preventive aspect against demineralization since it protects and lubricates oral surfaces and facilitates the cleaning of bacterial substrates [15]. Consequently, reduced salivary flow can diminish the preventive capacity of saliva [14, 15]. However, a recent review recognized that there is a need to design further studies to make a reliable assessment between hyposalivation and dental caries in young patients [16].

Prathima et al. study (2021) showed that maximum peak rise in salivary pH was observed immediately after spitting the CPP-ACP containing gums [17]. However, Ozdas et al. (2015) showed that the increase buffering capacity of CPP-ACP was observed since the fourth week of application [18] A recent systematic review showed that there is an evidence regarding the remineralizing effect of CPP-ACP by studying the roughness of enamel. However, the effect on physicochemical properties of saliva has not been studied in this review [19].

In developing countries such as Syria, parents do not give priority to the dental health of their children until they complain of pain [20]. In this phase, dental caries may progress into the irreversible stage in which remineralization protocols cannot be implemented [21].

There is a need to adopt protective protocols and apply materials that can enhance remineralization among Syrian children. A recent systematic review has indicated to the limited evidence about the cariostatic activity of CPP-ACP and addressed the need for further clinical trials [22]. Furthermore, the effect of CPP-ACP on physiochemical salivary properties is still controversial. More evidence to support the hypothesis that CPP-ACP can increase salivary flow rate and salivary pH, which in turn can enable remineralization, is still required. Therefore, this clinical trial was undertaken to investigate the changes of salivary flow rate and salivary pH of Syrian children with mixed dentition following application of CPP-ACP.

## Materials and methods

### Trial design

This study was a double-blind randomized controlled clinical trial conducted on 50 children aged 6–8 years. They were recruited from the Ambulance Charity Orphanage, Damascus, Syria. This trial was in accordance with the ethical standards of the institutional and national research committee of the study institution and with the 1964 Helsinki declaration and its later amendments or comparable ethical standards. It was approved by the Research Ethics Committee of Faculty of Dentistry of Damascus University and was registered at the ISRCTN registry (10.1186/ISRCTN17509082).

This randomized controlled clinical trial was designed, conducted and reported according to the CONSORT statement over a period of 7 months https://www.equator-network.org/reporting-guidelines/consort/.

The trial was a double blinded one in which the patients and the researcher had no idea about the agent that has been used. A random allocation list was designed using the website https://www.randomizer.org/ as computer-based methods for simple randomization are more reliable and easier to use than other manual methods.

### Sampling

Sample size was calculated according to previous similar study using the G*Power software (v. 3.1) (Franz Faul, Universitat Kiel, Germany). The significant level was set at *P* value of less than 0.05, and statistical power of study was set at 85%. It was considered that 50 patients were sufficient to demonstrate an effect size of 0.7. Therefore, 50 children aged between 6–8 years were selected in this study. All children who had good oral hygiene, (scored 0 according to Oral hygine Index OHI) [23], not taking antibiotics or any kind of medications for more than two weeks that can affect the flow rate of saliva were included. In addition, The inclusion criteria addressed that all particiapnts should not have any dental caries (code 0 according to international caries detection and assessment system ICDAS II) [24], any confirmed or suspected allergy to milk protein and/or the presence of the sensitivity of benzoate (preservative). All children who had diseases that may affect the flow rate of saliva such as diabetes were also excluded from study. Moreover, all children with orthodontic appliances were excluded.

### Study groups

**Fig. 1:**
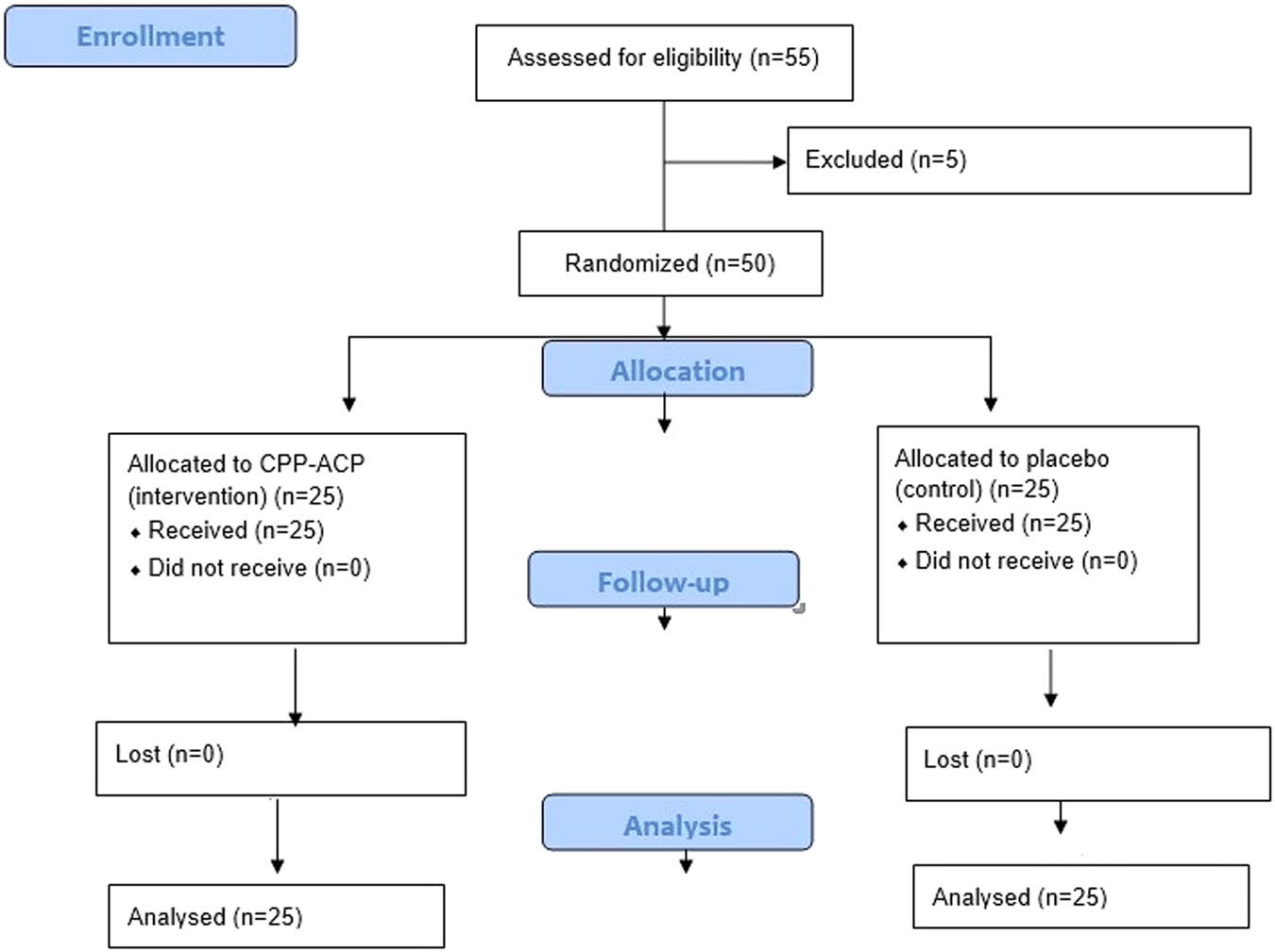
CONSORT flow diagram of participants. Diagram presents particiapnt flow through each stage of the study(enrollment, allocation, and analysis).

After full explanation about the aim of the study, written informed consent from the manager of the orphanage and their carers was obtained. All children recruited in this trial were volunteers who accepted to take part in this study. All children who met the inclusion criteria were equally and randomly assigned into either Group A which included 25 children who had CPP-ACP (GC Tooth Mousse™) application (Intervention), or Group B that contained 25 children who received placebo mousse as controls. Five children were excluded as they were not cooperative. Figure 1 shows the CONSORT flow diagram of the participants.

### Chemical prepaeration of the placebo cream

The placebo cream was prepared by the researcher in the Department of Pharmaceutical Industries at the Faculty of Pharmacy, Damascus University. The prepared placebo cream mimics the intervention mousse (GC tooth Mousse™) with the physical characteristics (color, flavor and texture) in order to help in the blinding process.

### Topical application of the studied mousse

Supervisors of the orphanage were asked to brush the children’s teeth with water only on the day before the experiment. A thick layer of the material was applied within the upper and lower trays for 3 min. Then, after removing the tray, child was asked to distribute the remaining material on the teeth by the tongue without swallowing it. This method of application was used for both groups in this trial and the period was standardized to two minutes for all children. Children were asked to dispose of the substance, but without rinsing the mouth. They were also asked not to eat or drink until the end of the saliva sampling period.

### Clinical intervention and saliva collection

Salivary samples were collected, by the principal investigator LA, in the morning between 08:00 and 10:00 am. At the first stage, children were given paraffin wax to be chewed and they were asked to swallow the pooled saliva. Then, they were asked to spit their saliva into saliva collecting glass tube for five minutes. The amount of collected saliva was then calculated and divided into 5 in order to report the amount of saliva collected per minute (ml/m). This process was performed four times: before the application of the studied agents (T0), directly after the application (T1), after half an hour (T2) and after one hour (T3). Salivary flow rate was measured at four time points. In addition, pH of saliva was measured in each time point using pH test strips (Saliva-Check Buffer, GC Int). The pH test strips were soaked within saliva for one minute. Accurate color change indicating pH values was observed and compared with the accompanying chromatography table.

### Data analysis

Data were analyzed using IBM SPSS version 23 (IBM Corp., Armonk, USA). Descriptive data, including minimum, maximum, mean, and standard deviation, were calculated for the studied groups. Shapiro-Wilk test of normality showed that data were normally distributed. Two-way repeated measures ANOVA test was used to test the differences existed between the two groups and different time points regarding the saliva pH and salivary flow rate with Bonferroni Test post hoc application for multiple comparisons. Greenhouse–Geisser correction test was applied when the assumption of the sphericity of Mauchly’s test is violated. The two-tailed probability value of *P* < 0.05 was considered statistically significant.

## Results

The research sample included 50 children (30 boys and 20 girls), who were distributed according to the random distribution tables and categorized into two groups in which 25 children were included in each group.

The mean values for the salivary flow rate for both groups at T0, T1, T2, and T3 were 0.41 ± 0.30, 0.65 ± 0.36, 0.53 ± 0.28, and 0.56 ± 0.34 respectively. The findings of the independent *t-*test indicated that there was no significant difference between group A and B in the mean value of salivary flow rate at bassline (*t* = 1.08, *P* = 0.28, 0.57 ± 0.28 versus 0.56 ± 0.38 respectively). However, the one-way ANOVA test indicated to the presence of significant difference between different time points in the mean value of salivary flow rate within groups (*F* = 4.5, df = 196, *P* = 0.004). According to Bonferroni post hoc test, which assessed the effects of treatment and time, salivary flow rate increased significantly in T1 and then decreased significantly. The findings are presented in Table 1.

**Table 1:** One way ANOVA for salivary flow rate analysis in different time points.

Group	T0	T1	T2	T3	F	df	*P*-value
A	0.40	0.68	0.54	0.57	4.5	196	0.004
B	0.43	0.62	0.52	0.55	4.3	187	0.004

In addition, the mean values for the salivary pH, for both groups, at T0, T1, T2, and T3 were 6.99 ± 0.44, 7.46 ± 0.36, 7.36 ± 0.32, and 7.26 ± 0.32 respectively. There was no significant difference between group A and B in the mean value of salivary pH (*t* = 0.61, *P* = 0.54, 7.28 ± 0.44 versus 7.25 ± 0.36 respectively). However, the one-way ANOVA indicated to the presence of significant difference between different time points in the mean value of salivary pH within groups (*F* = 15.23, df = 196, *P* = 0,000). According to Bonferroni post hoc test, which assessed the effects of treatment and time, the salivary pH increased dramatically in T1 and then decreased significantly. Descriptive data of both salivary pH and salivary flow rate for all children in each time point are presented in Table 2.

**Table 2:** Descriptive data of both salivary pH and salivary flow rate.

Measures	Group	Mean	Standard deviation	Minimum	Maximum
Saliva pH	A	7.25	0.432	6	8
	B	7.28	0.456	6.52	7.88
Salivary flow rate	A	0.55	0.336	0.10	2.24
	B	0.52	0.320	0.09	2.19

Two-way repeated measures ANOVA test showed that the difference between the two treatments was not significant in the mean value of saliva pH (F = 0.92, df = 1, *P* = 0.346). However, there was a statistically significant difference (*F* = 29.861, df = 3, *P* = 0.00) between the different time points in the mean value of saliva pH. In addition, the two-way repeated measures ANOVA test indicated that there was no statistically significant difference between group A and B (*F* = 0.066, df = 1, *P* = 0.211) in the rate of salivary flow although the difference was statistically significant (*F* = 11.74, df = 3, *P* = 0.000) between the time points (Table 3).

**Table 3:** Findings of the two way repeated measures ANOVA test to analyze the difference between the time points in each group regarding the two variables.

Variable	Group	T0	T1	T2	T3	*F*-value	*P*-value
Saliva PH	A	6.88 ± 0.415	7.50 ± 0.32	7.34 ± 0.37	7.26 ± 0.326	1.226	0.001^a^
	B	7.12 ± 0.46	7.46 ± 0.406	7.4 ± 0.25	7.30 ± 0.353	1.425	0.002^a^
Salivary flow rate	A	0.34 ± 0.26	0.68 ± 0.334	0.57 ± 0.274	0.54 ± 0.207	1.001	0.000^a^
	B	0.51 ± 0.34	0.62 ± 0.388	0.49 ± 0.3	0.58 ± 0.451	0.993	0.000^a^

^a^Statistically significant difference.

**Fig. 2:**
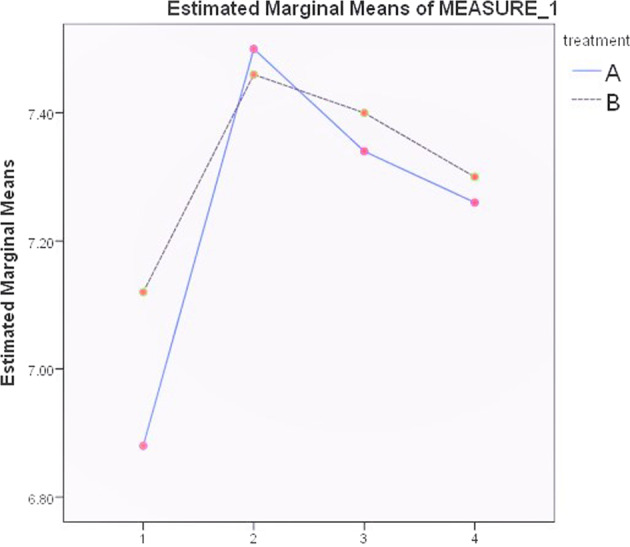
The change of saliva pH values of groups A and B in the studied time points. (X-axis: T0,T1,T2 and T3; Y-axis: pH values).

**Fig. 3:**
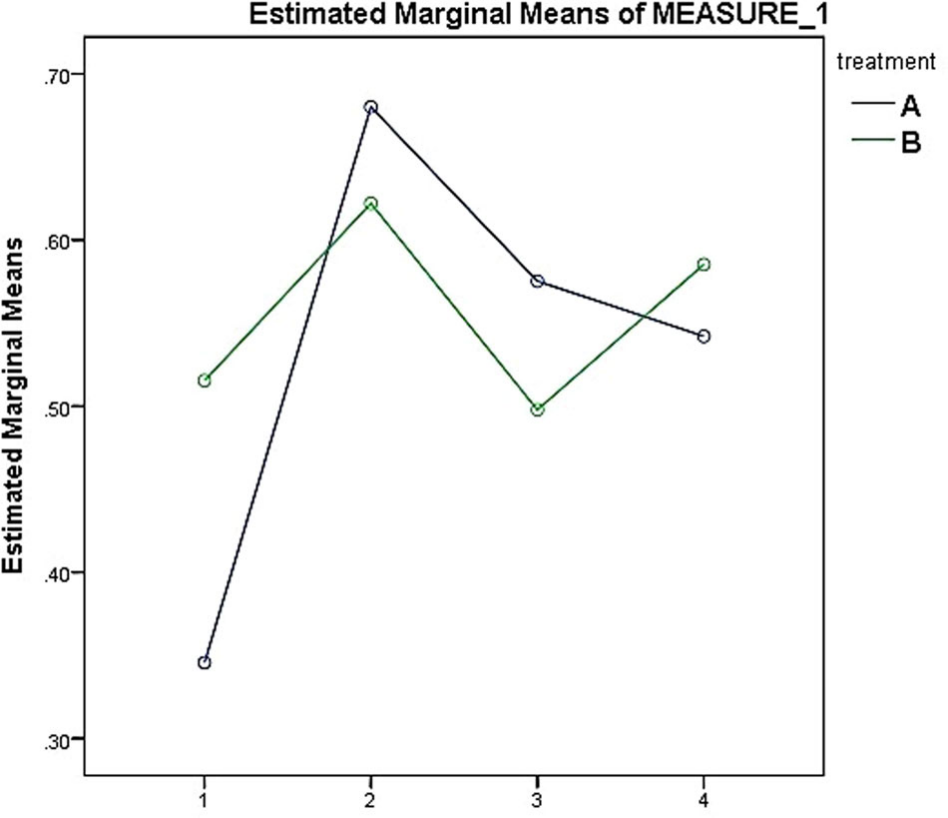
The change of salivary flow rate values of groups A and B in the studied time points. (X-axis: T0,T1,T2 and T3; Y-axis: salivary flow rate values [ml/min]).

Multiple comparisons with baseline using Bonferroni correction showed significant differences in the mean value of saliva pH of group A over time from baseline to T1 and then a significant decrease (*P* < 0.001) from T2 to T3 (Fig. 2). In addition, multiple comparisons with baseline using Bonferroni correction showed significant differences in the salivary flow rate of group A from baseline to T1 and then a significant decrease(*P* < 0.001) fromT2 to T3 (Fig. 3).

The two-way repeated ANOVA using a mixed model between subject and within-subject factors revealed that Mauchly’s test of sphericity has been met for the rate of salivary flow (*x*^2^ = 5.58, *P* > 0.05) but not for salivary pH(*x*^*2*^ = 11.338, *P* = 0.04). There was no significant difference in the rate of salivary flow and salivary pH between two groups over time. However, variances between group A and B were significantly different from one another based on an alpha of 0.05, *P* < 0.001, indicating the sphericity assumption was violated for time (Table 4). Therefore, the Greenhouse–Geisser correction was applied for multivariate analysis. The correction showed that there were no statistically significant effects or changes between group A and B over time in the rate of salivary flow and saliva pH. This means that there was no significant difference in the efficacy of CPP-ACP on salivary flow rate and pH compared to the control group (Table 4).

**Table 4:** Mauchly’s test of sphericity

Variable	Within subjects effect	Mauchly’s W	Approx. Chi-Square	df	Sig.
Saliva pH	Treatment group	1	0.000	0	0.000*
	Time	0.734	7.020	5	0.220
	Treatment* Time	0.61	11.338	5	0.04
Salivary flow rate	Treatment group	1	0.000	0	0.000*
	Time	0.775	5.783	5	0.328
	Treatment* Time	0.782	5.585	5	0.349

*P value is less than 0.05.

## Discussion

CPP-ACP is a compound developed to enable remineralization by generating a pool of calcium and phosphate which maintain the supersaturation of saliva [11, 25, 26]. Previous studies provided controversial results for the influence of CPP-ACP on physiochemical properties of saliva. For that reason, it is of interest to establish whether the application of CPP-ACP can raise the saliva pH or increase the salivary flow rate in children and consequently contribute to remineralization. To the best of our knowledge, this trial is the first to study the impact of topical application of CPP-ACP for a short period on the saliva pH and salivary flow rate in children.

Previous studies indicated that CPP-ACP could be used to inhibit demineralization and to improve remineralization. For instance, a recent review (2020) showed that CPP-ACP could be an effective agent to treat white spot lesions around orthodontic brackets. However, they found that the remineralization effect of CPP-ACP is not significantly greater than using Fluoride alone [27]. This can be referred to what have mentioned Ferrazzano et al. (2011) that the effect of CPP-ACP on demineralized enamel is affected by many elements in the oral cavity that are difficult to control [28].

Similarly, the study of Shen et al. (2021) found that the presence of saliva and biofilm is crucial to authenticate the mechanism of action of CPP-ACP in remineralization [29, 30]. The amount of secreted saliva and saliva pH can both alter the way CPP-ACP remineralizes tooth enamel as CPP separates from ACP in the acidic environment [28, 29].

In the present trial, 50 children aged 6 to 8 years old were selected since this age group has been at high risk of caries due to the cariogenic diet they consume[31]. In addition, a limited age range was selected to avoid the age-related differences in physiochemical properties of saliva.

To standardize the diet intake of the participants, all children recruited in this trial were living in one orphanage. Stimulated saliva was selected to be studied in this trial in different time points before and after the application of CPP-ACP because stimulated saliva is more related with caries prevention than unstimulated saliva [31, 32]. This is due to the higher mineral content that leads to more buffering capacity and greater salivary clearance [32–34].

In this study, saliva samples were collected in the morning (08:00–10:00 am) to minimize the effect of circadian variation in the salivary flow rate. saliva-check Buffer kit (Saliva-Check Buffer, GC Int) was used to check the salivary pH, as it was an easy to use, and reliable method [35]. In addition, the salivary pH was measured immediately after the determined contact time of the color-changing strip (10 s) to avoid potential misreading of the results. In this trial, there was no significant difference between the two intervention groups regarding the salivary flow rate. This may be attributed to the ability of the placebo cream to stimulate salivary secretion simply due to introducing a new agent into the oral cavity, considering the presence of any substance in the oral cavity induces both chemical and olfactory stimuli by neural reflexes, which leads to raised output of saliva.

Our results were different from those reported by Hegde and Thakkar(2017) which showed that CPP-ACP can increase the salivary flow rate [36]. This variation in the results can be due to the use of chewing gum in their study, which may increase the flow rate of saliva regardless of its composition. However, our results were in accordance with another study, which indicated that the topical application of CPP-ACP did not alter the salivary flow rate [37].

Moreover, this study showed that there was no significant difference between the two groups in the changes of saliva pH. This can be explained by the results of previous studies which indicated that CPP-ACP can increase salivary pH after a long period [38]. For instance, Ozdas et al. (2015) showed that the increase buffering capacity of CPP-ACP was observed since the fourth week of application [18]. Therefore, our results were different from the results of Emamieh et al. (2015) which showed that CPP-ACP containing chewing gum can increase the level of saliva pH [39]. This difference may be attributed to the duration of chewing the gum over three weeks that was assessed during the trial.

Padminee et al. (2018) reported that CPP-ACP products could raise the pH of saliva more than xylitol. They attributed that results to the ability of CPP-ACP in bringing down *Streptococcus mutans* levels which are acidogenic pathogens [40].

The present findings indicated that both salivary flow rate and saliva pH were increased directly after the application of both placebo and CPP-ACP with a statistically significant difference between different time points. These findings were in agreement with of Prathima et al. study (2021) which showed that maximum peak rise in salivary pH was observed immediately after spitting the CPP-ACP containing gums [17] de Oliveira PRA et al. (2022) showed in their study that tooth remineralization of initial teeth demineralization gives better results in the presence of fluoride in conjunction with CPP-ACP. They also reported that placebo-treated specimens incorporated fluoride from the saliva due to the high sensitivity of white spot lesions (demineralized enamel) to pH cycling. For that reason, they found that remineralization process would be better achieved in the presence of fluoride dentifrice [7].

In fact, it should be emphasized that this study has highlighted the importance of investigating the remineralization products such as CPP-ACP for a short period among children, which might be a limitation. However, further studies should investigate the effect of CPP-ACP on a longer period of application to ascertain findings. In addition, the study looked at two elements of CCP-ACP including salivary flow rate and salivary pH. It is possible that CPP-ACP is effective in remineralization by a mechanism separate to the salivary flow rate or salivary pH. Future work should consider other parameters such as the measurement of calcium concentration, which might provide further understanding about the effect of CPP-ACP in remineralization process.

## Conclusion

This study has supported the null hypothesis that single topical application of the GC Tooth Mouse (CPP-ACP) has no effect on increasing the salivary pH and salivary flow rate. Topical application of CPP-ACP can raise the level of saliva pH and the salivary flow rate immediately after the application for a short period of less than half an hour. Further studies are still important with increased sample size to ascertain findings.

## Data Availability

The datasets used and/or analyzed during the current study are available from the corresponding author on reasonable request.

## References

[1] Pitts NB, Zero DT, Marsh PD, Ekstrand K, Weintraub JA, Ramos-Gomez F (2017). Dental caries. Nat Rev Dis Prim..

[2] Frencken JE, Sharma P, Stenhouse L, Green D, Laverty D, Dietrich T (2017). Global epidemiology of dental caries and severe periodontitis–a comprehensive review. J Clin Periodontol..

[3] Cochrane NJ, Cai F, Huq NL, Burrow MF, Reynolds EC (2010). New approaches to enhanced remineralization of tooth enamel. J Dent Res..

[4] Murtazaev SS, Akhrorkhujaev NS, Kiselnikova LP, Dinikulov JA, Astanakulova MM (2020). Oral health and prevention of dental caries in preschool children living in conditions of biogeochemical fluorine deficiency. Eur J Mol Clin Med..

[5] AlFeel J, Laflouf M, AlKurdi S, Alkhouli M (2021). Evaluating the effect of Clinpro Tooth Crème on remineralization of pre-carious White Spot Lesions in anterior primary teeth: randomized controlled clinical trial. Pediatr Dent J..

[6] Ten Cate JM (2012). Novel anticaries and remineralizing agents: prospects for the future. J Dent Res..

[7] de Oliveira PRA, Barreto LSDC, Tostes MA (2022). Effectiveness of CPP‐ACP and fluoride products in tooth remineralization. Int J Dent Hyg..

[8] Gupta R, Prakash V (2011). CPP-ACP complex as a new adjunctive agent for remineralisation: a review. Oral Heal Prev Dent..

[9] Fernando JR, Shen P, Sim CPC, Chen YY, Walker GD, Yuan Y (2019). Self-assembly of dental surface nanofilaments and remineralisation by SnF2 and CPP-ACP nanocomplexes. Sci Rep..

[10] Huq NL, Myroforidis H, Cross KJ, Stanton DP, Veith PD, Ward BR (2016). The interactions of CPP–ACP with saliva. Int J Mol Sci..

[11] Oshiro M, Yamaguchi K, Takamizawa T, Inage H, Watanabe T, Irokawa A (2007). Effect of CPP-ACP paste on tooth mineralization: an FE-SEM study. J Oral Sci..

[12] Farsi N (2008). Dental caries in relation to salivary factors in Saudi population groups. J Contemp Dent Pr.

[13] Sekhri P, Sandhu M, Sachdev V, Chopra R (2018). Estimation of trace elements in mixed saliva of caries free and caries active children. J Clin Pediatr Dent.

[14] González-Aragón Pineda AE, García Pérez A, García-Godoy F (2020). Salivary parameters and oral health status amongst adolescents in Mexico. BMC Oral Health.

[15] Do T, Damé-Teixeira N, Naginyte M, Marsh PD. Root surface biofilms and caries. In: Root caries: from prevalence to therapy. São Paulo: Karger Publishers, 2017, pp. 26–34. 10.1159/000479304 29050018

[16] dos Santos Letieri A, Siqueira WL, Solon-de-Mello M, Masterson D, Freitas-Fernandes LB, Valente AP, et al. A critical review on the association of hyposalivation and dental caries in children and adolescents. Arch Oral Biol. 2022;144:105545. 10.1016/j.archoralbio.2022.105545 36209541

[17] Prathima GS, Narmatha M, Selvabalaji A, Adimoulame S, Ezhumalai G (2021). Effects of Xylitol and CPP-ACP chewing gum on salivary properties of children with molar incisor hypomineralization. Int J Clin Pediatr Dent.

[18] Özdas DÖ, Tuna EB, Yilmaz EY, Aytepe Z (2015). Casein phosphopeptide-amorphous calcium phosphate (CPP-ACP) may be an alternative preventive therapy in children with cerebral palsy. Oral Heal Prev Dent.

[19] Ma X, Lin X, Zhong T, Xie F (2019). Evaluation of the efficacy of casein phosphopeptide-amorphous calcium phosphate on remineralization of white spot lesions in vitro and clinical research: a systematic review and meta-analysis. BMC Oral Health.

[20] Dashash M, Blinkhorn A (2012). The dental health of 5 year-old children living in Damascus, Syria. Community Dent Health.

[21] Ballouk MA-H, Dashash M (2019). Caries prevalence and dental health of 8–12 year-old children in Damascus city in Syria during the Syrian Crisis; a cross-sectional epidemiological oral health survey. BMC Oral Health.

[22] Singal K, Sharda S, Gupta A, Malik VS, Singh M, Chauhan A, et al. Effectiveness of Calcium Phosphate derivative agents on the prevention and remineralization of caries among children-A systematic review & meta-analysis of randomized controlled trials. J Evid Based Dent Pract 2022;22:101746. 10.1016/j.jebdp.2022.101746 36162884

[23] Kokoceva-Ivanovska OR, Sarakinova O, Zabokova-Bilbilova E, Mijoska AN, Stavreva N (2018). Oral hygiene index in early childhood caries, before and after topical fluoride treatment. Open Access Maced J Med Sci.

[24] Ekstrand KR, Gimenez T, Ferreira FR, Mendes FM, Braga MM (2018). The international caries detection and assessment system–ICDAS: a systematic review. Caries Res.

[25] Sionov RV, Tsavdaridou D, Aqawi M, Zaks B, Steinberg D, Shalish M (2021). Tooth mousse containing casein phosphopeptide-amorphous calcium phosphate prevents biofilm formation of Streptococcus mutans. BMC Oral Health.

[26] Yengopal V, Mickenautsch S (2009). Caries preventive effect of casein phosphopeptide-amorphous calcium phosphate (CPP-ACP): a meta-analysis. Acta Odontol Scand..

[27] Wang Y, Hua F, Jiang H (2020). CPP-ACP may be effective, but not significantly greater than using fluorides alone, in preventing and treating white spot lesions around orthodontic brackets. J Evid Based Dent Pract..

[28] Ferrazzano GF, Amato I, Cantile T, Sangianantoni G, Ingenito A (2011). In vivo remineralising effect of GC tooth mousse on early dental enamel lesions: SEM analysis. Int Dent J..

[29] Shen P, Fernando JR, Yuan Y, Walker GD, Reynolds C, Reynolds EC (2021). Bioavailable fluoride in calcium-containing dentifrices. Sci Rep..

[30] Walsh T, Worthington HV, Glenny A, Marinho VC, Jeroncic A. Fluoride toothpastes of different concentrations for preventing dental caries. Cochrane Database Syst Rev. 2019;3:CD007868. 10.1002/14651858.CD007868.pub3 PMC6398117 30829399

[31] Van Chuyen N, Van Du V, Van Ba N, Long DD, Son HA (2021). The prevalence of dental caries and associated factors among secondary school children in rural highland Vietnam. BMC Oral Health.

[32] Abufarwa M, Noureldin A, Dziak R, Covell D (2022). Efficacy of CPP-ACP fluoride varnish applied with and without acid etching in preventing enamel demineralization compared to light-curable fluoride varnish. Angle Orthod.

[33] Kapourani A, Kontogiannopoulos KN, Manioudaki A-E, Poulopoulos AK, Tsalikis L, Assimopoulou AN (2022). A review on xerostomia and its various management strategies: the role of advanced polymeric materials in the treatment approaches. Polymers.

[34] Nederfors T (2000). Xerostomia and hyposalivation. Adv Dent Res..

[35] Song C-W, Kim H-K, Kim M-E (2015). Clinical usefulness of pH papers in the measurement of salivary pH. J Oral Med Pain.

[36] Hegde RJ, Thakkar JB (2017). Comparative evaluation of the effects of casein phosphopeptide-amorphous calcium phosphate (CPP-ACP) and xylitol-containing chewing gum on salivary flow rate, pH and buffering capacity in children: an in vivo study. J Indian Soc Pedod Prev Dent..

[37] Peric T, Markovic D, Petrovic B, Radojevic V, Todorovic T, Radicevic BA (2015). Efficacy of pastes containing CPP-ACP and CPP-ACFP in patients with Sjögren’s syndrome. Clin Oral Investig.

[38] Bakkal M, Abbasoglu Z, Kargul B (2017). The effect of casein phosphopeptide-amorphous calcium phosphate on molar-incisor hypomineralisation: a pilot study. Oral Heal Prev Dent.

[39] Emamieh S, Khaterizadeh Y, Goudarzi H, Ghasemi A, Baghban AA, Torabzadeh H (2015). The effect of two types chewing gum containing casein phosphopeptide-amorphous calcium phosphate and xylitol on salivary Streptococcus mutans. J Conserv Dent JCD.

[40] Padminee K, Poorni S, Diana D, Duraivel D, Srinivasan MR (2018). Effectiveness of casein phosphopeptide-amorphous calcium phosphate and xylitol chewing gums on salivary pH, buffer capacity, and Streptococcus mutans levels: an interventional study. Indian J Dent Res..

